# Crystal structure of 8-hy­droxy­quinolin­ium 2-carboxy-6-nitro­benzoate mono­hydrate

**DOI:** 10.1107/S205698901500571X

**Published:** 2015-03-25

**Authors:** M. Divya Bharathi, G. Ahila, J. Mohana, G. Chakkaravarthi, G. Anbalagan

**Affiliations:** a Department of Physics, Presidency College, Chennai 600 005, India; b Department of Physics, CPCL Polytechnic College, Chennai 600 068, India

**Keywords:** crystal structure, 8-hy­droxy­quinolinium, 2-carboxy-6-nitro­benzoate, hydrogen bonding, π–π inter­actions

## Abstract

In the title hydrated salt, C_9_H_8_NO^+^·C_8_H_4_NO_6_^−^·H_2_O, the deprotonated carboxyl­ate group is almost normal to its attached benzene ring [dihedral angle = 83.56 (8)°], whereas the protonated carboxyl­ate group is close to parallel [dihedral angle = 24.56 (9)°]. In the crystal, the components are linked by N—H⋯O and O—H⋯O hydrogen bonds, generating [001] chains. The packing is consolidated by C—H⋯O and π–π [centroid-to-centroid distances = 3.6408 (9) and 3.6507 (9) Å] inter­actions, which result in a three-dimensional network.

## Related literature   

For the biological activity of quinoline derivatives, see: Font *et al.* (1997 ▸); Sloboda *et al.* (1991 ▸). For similar structures, see: Castañeda *et al.* (2014 ▸); Kafka *et al.* (2012 ▸); Li & Chai (2007 ▸).
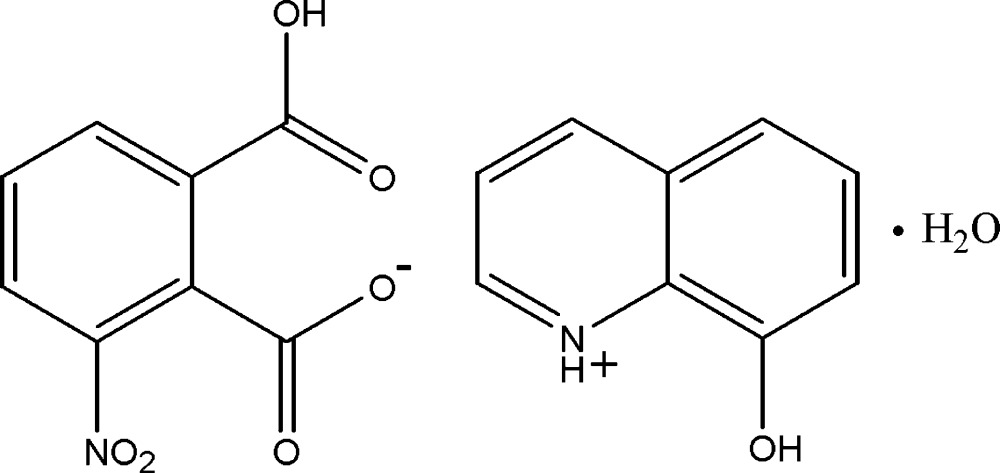


## Experimental   

### Crystal data  

C_9_H_8_NO^+^·C_8_H_4_NO_6_^−^·H_2_O
*M* *_r_* = 374.30
Monoclinic, 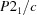

*a* = 14.4283 (5) Å
*b* = 13.8196 (5) Å
*c* = 8.0483 (3) Å
β = 101.441 (2)°
*V* = 1572.89 (10) Å^3^
*Z* = 4
Mo *K*α radiation
μ = 0.13 mm^−1^
*T* = 295 K
0.26 × 0.22 × 0.18 mm

### Data collection  

Bruker Kappa APEXII CCD diffractometer
Absorption correction: multi-scan (*SADABS*; Sheldrick, 1996 ▸) *T*_min_ = 0.968, *T*_max_ = 0.977
58922 measured reflections
7431 independent reflections
4272 reflections with *I* > 2σ(*I*)
*R*_int_ = 0.034

### Refinement  

*R*[*F* ^2^ > 2σ(*F* ^2^)] = 0.060
*wR*(*F* ^2^) = 0.176
*S* = 1.02
7431 reflections
260 parameters
5 restraints
H atoms treated by a mixture of independent and constrained refinement
Δρ_max_ = 0.50 e Å^−3^
Δρ_min_ = −0.38 e Å^−3^

### 

Data collection: *APEX2* (Bruker, 2004 ▸); cell refinement: *SAINT* (Bruker, 2004 ▸); data reduction: *SAINT*; program(s) used to solve structure: *SHELXS97* (Sheldrick, 2008 ▸); program(s) used to refine structure: *SHELXL97* (Sheldrick, 2008 ▸); molecular graphics: *PLATON* (Spek, 2009 ▸); software used to prepare material for publication: *SHELXL97*.

## Supplementary Material

Crystal structure: contains datablock(s) global, I. DOI: 10.1107/S205698901500571X/hb7385sup1.cif

Structure factors: contains datablock(s) I. DOI: 10.1107/S205698901500571X/hb7385Isup2.hkl

Supporting information file. DOI: 10.1107/S205698901500571X/hb7385Isup3.cml

**Fig. 1.**
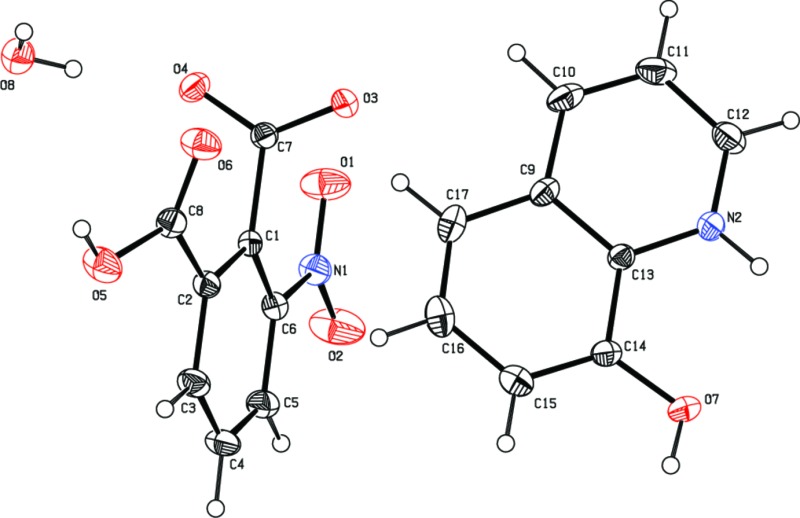
The molecular structure of (I), with 30% probability displacement ellipsoids for non-H atoms.

**Fig. 2.**
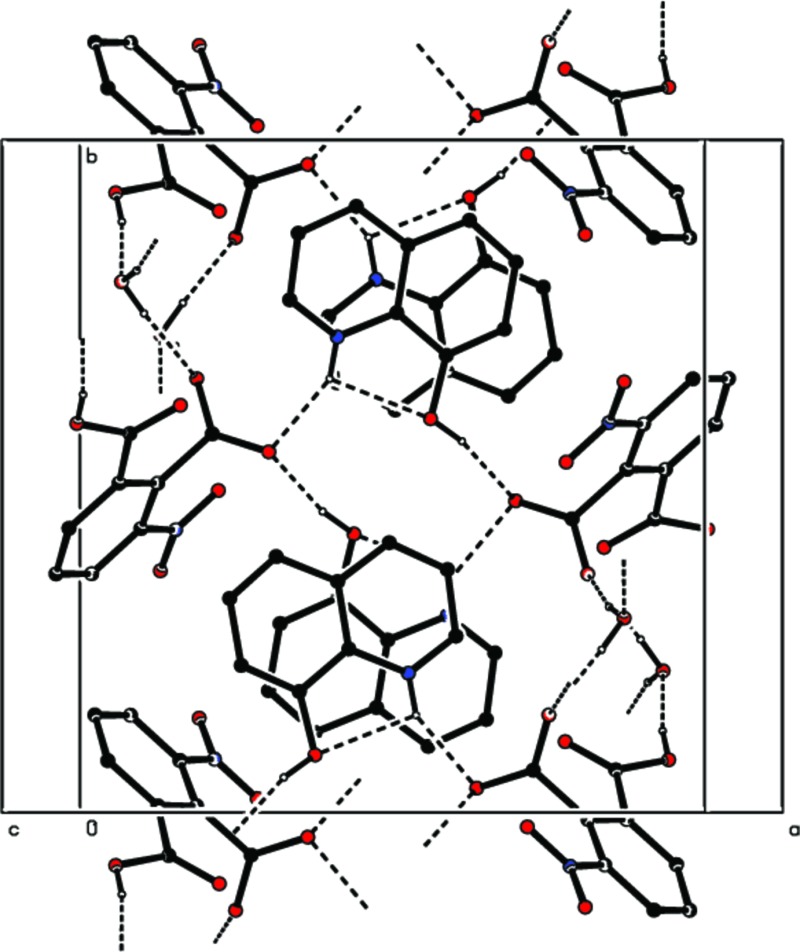
The packing of (I), viewed down c axis. Hydrogen bonds are shown as dashed lines. H atoms not involved in hydrogen bonding have been omitted.

. DOI: 10.1107/S205698901500571X/hb7385fig1.tif
The mol­ecular structure of (I), with 30% probability displacement ellipsoids for non-H atoms.

c . DOI: 10.1107/S205698901500571X/hb7385fig2.tif
The packing of (I), viewed down *c* axis. Hydrogen bonds are shown as dashed lines. H atoms not involved in hydrogen bonding have been omitted.

CCDC reference: 1055171

Additional supporting information:  crystallographic information; 3D view; checkCIF report

## Figures and Tables

**Table 1:** Hydrogen-bond geometry (, )

*D*H*A*	*D*H	H*A*	*D* *A*	*D*H*A*
N2H2O3^i^	0.89(1)	2.00(1)	2.8112(16)	151(2)
O5H5*A*O8^ii^	0.82(1)	1.78(1)	2.5928(18)	171(3)
O7H7O3^iii^	0.84(1)	1.82(1)	2.6482(15)	168(2)
O8H8*B*O4	0.83(1)	2.07(1)	2.8683(17)	163(2)
O8H8*A*O4^ii^	0.83(1)	2.01(1)	2.8288(18)	170(2)
C11H11O1^iv^	0.93	2.42	3.295(2)	156
C12H12O6^i^	0.93	2.48	3.343(2)	155
C16H16O2^v^	0.93	2.52	3.413(2)	160

Symmetry codes: (i) 
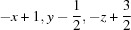
; (ii) 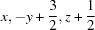; (iii) 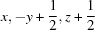; (iv) 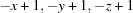; (v) 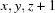.

## supplementary crystallographic information

### S1. Chemical context

The quinoline nucleus is found in many synthetic and natural products having a wide range of pharmacological activities such as anti-viral (Font *et al.*, 1997), and anti-inflammatory (Sloboda *et al.*, 1991) activities.

### S2. Structural commentary

We herewith report the crystal structure of the title compound (I), (Fig.1). The asymmetric unit of the title compound consists of C_9_ H_8_ N O^+^ cation, C_8_ H_4_ N O_6_^-^ anion and a water molecule. The geometric parameters of the title compound are comparable to the reported structures [Castañeda *et al.*, 2014; Kafka *et al.*, 2012; Li & Chai (2007)]. The benzene ring (C1—C6) of anion makes the dihedral angle of 58.18 (6)° with the quinolinium ring (C9—C12/N2/C13—C17) of cation.

### S3. Supra­molecular features

The molecular structure is stabilized by weak intra­molecular N—H···O and O—H···O hydrogen bonds (Table 1). The crystal structure is formed by weak inter­molecular N—H···O, O—H···O and C—H···O hydrogen bonds (Table 1 & Fig. 2) by linking the adjacent anions and cations by bridging water molecules through O—H···O hydrogen bonds into infinite two-dimensional network along [1 0 0] plane. The crystal structure is further stabilized by weak C—H···π (Table 1) and π–π [Cg1···Cg1^i^ = 3.6507 (9); Cg2···Cg2^ii^ = 3.6507 (9)Å; (i) -x,1-y,1-z; (ii) x,1/2-y,1/2+z; Cg1 and Cg2 are the centroids of the rings (C1—C6) and (N2/C12/C11/C10/C9/C13)] inter­actions.

### S4. Synthesis and crystallization

The title compound was synthesized by taking at 1:1 ratio of 8-hy­droxy­quinoline and of 3-nitro­phthalic acid was dissolved in a mixed solvent of methanol and water. The salt was formed while adding the base instanstaouly. The solution was stirred for about 2 h to get a homogenous solution. The solution was filtered off and kept aside for slow evaporation at room temperature which yields single crystals suitable for X-ray diffraction.

### S5. Refinement

C-bound H atoms were positioned geometrically and refined using riding model with C—H = 0.93 Å and Uiso(H) = 1.2Ueq(C). H atoms for O atoms were located from Fourier map and refined with O—H = 0.82 (1)Å and Uiso(H) = 1.5 Ueq(O). H atom for N atom was located from Fourier map and refined freely with N—H = 0.88 (1)Å.

### Crystal data

**Table:**

C_9_H_8_NO^+^·C_8_H_4_NO_6_^−^·H_2_O	*F*(000) = 776
*M**_r_* = 374.30	*D*_x_ = 1.581 Mg m^−^^3^
Monoclinic, *P*2_1_/*c*	Mo *K*α radiation, λ = 0.71073 Å
Hall symbol: -P 2ybc	Cell parameters from 9900 reflections
*a* = 14.4283 (5) Å	θ = 2.8–33.4°
*b* = 13.8196 (5) Å	µ = 0.13 mm^−^^1^
*c* = 8.0483 (3) Å	*T* = 295 K
β = 101.441 (2)°	Block, colourless
*V* = 1572.89 (10) Å^3^	0.26 × 0.22 × 0.18 mm
*Z* = 4	

### Data collection

**Table:**

Bruker Kappa APEXII CCD diffractometer	7431 independent reflections
Radiation source: fine-focus sealed tube	4272 reflections with *I* > 2σ(*I*)
Graphite monochromator	*R*_int_ = 0.034
ω and φ scans	θ_max_ = 36.1°, θ_min_ = 2.1°
Absorption correction: multi-scan (*SADABS*; Sheldrick, 1996)	*h* = −23→23
*T*_min_ = 0.968, *T*_max_ = 0.977	*k* = −19→22
58922 measured reflections	*l* = −13→12

### Refinement

**Table:**

Refinement on *F*^2^	Primary atom site location: structure-invariant direct methods
Least-squares matrix: full	Secondary atom site location: difference Fourier map
*R*[*F*^2^ > 2σ(*F*^2^)] = 0.060	Hydrogen site location: inferred from neighbouring sites
*wR*(*F*^2^) = 0.176	H atoms treated by a mixture of independent and constrained refinement
*S* = 1.02	*w* = 1/[σ^2^(*F*_o_^2^) + (0.0619*P*)^2^ + 0.9848*P*] where *P* = (*F*_o_^2^ + 2*F*_c_^2^)/3
7431 reflections	(Δ/σ)_max_ < 0.001
260 parameters	Δρ_max_ = 0.50 e Å^−^^3^
5 restraints	Δρ_min_ = −0.38 e Å^−^^3^

### Special details

**Table:**

Geometry. All esds (except the esd in the dihedral angle between two l.s. planes) are estimated using the full covariance matrix. The cell esds are taken into account individually in the estimation of esds in distances, angles and torsion angles; correlations between esds in cell parameters are only used when they are defined by crystal symmetry. An approximate (isotropic) treatment of cell esds is used for estimating esds involving l.s. planes.
Refinement. Refinement of F^2^ against ALL reflections. The weighted R-factor wR and goodness of fit S are based on F^2^, conventional R-factors R are based on F, with F set to zero for negative F^2^. The threshold expression of F^2^ > 2sigma(F^2^) is used only for calculating R-factors(gt) etc. and is not relevant to the choice of reflections for refinement. R-factors based on F^2^ are statistically about twice as large as those based on F, and R- factors based on ALL data will be even larger.

### Fractional atomic coordinates and isotropic or equivalent isotropic displacement parameters (Å^2^)

**Table:**

	*x*	*y*	*z*	*U*_iso_*/*U*_eq_	
C1	0.17265 (9)	0.48978 (9)	0.55989 (18)	0.0237 (2)	
C2	0.13410 (10)	0.48988 (10)	0.70682 (19)	0.0262 (3)	
C3	0.06506 (11)	0.42272 (12)	0.7270 (2)	0.0331 (3)	
H3	0.0403	0.4238	0.8252	0.040*	
C4	0.03290 (11)	0.35481 (12)	0.6037 (2)	0.0370 (4)	
H4	−0.0117	0.3091	0.6203	0.044*	
C5	0.06716 (11)	0.35508 (12)	0.4557 (2)	0.0339 (3)	
H5	0.0449	0.3108	0.3702	0.041*	
C6	0.13531 (10)	0.42230 (10)	0.43632 (19)	0.0267 (3)	
C7	0.24827 (10)	0.56315 (10)	0.53939 (18)	0.0249 (3)	
C8	0.16432 (11)	0.56359 (11)	0.84160 (19)	0.0294 (3)	
C9	0.46422 (11)	0.34368 (10)	0.87844 (19)	0.0288 (3)	
C10	0.53122 (13)	0.40133 (12)	0.8201 (2)	0.0377 (4)	
H10	0.5225	0.4680	0.8125	0.045*	
C11	0.60883 (13)	0.36137 (14)	0.7744 (2)	0.0424 (4)	
H11	0.6521	0.4002	0.7341	0.051*	
C12	0.62252 (11)	0.26245 (14)	0.7886 (2)	0.0390 (4)	
H12	0.6752	0.2346	0.7575	0.047*	
C13	0.48139 (9)	0.24333 (10)	0.89029 (18)	0.0247 (3)	
C14	0.41699 (10)	0.18003 (10)	0.94525 (19)	0.0284 (3)	
C15	0.33963 (11)	0.21885 (13)	0.9934 (2)	0.0364 (3)	
H15	0.2978	0.1785	1.0346	0.044*	
C16	0.32192 (13)	0.31850 (15)	0.9818 (2)	0.0432 (4)	
H16	0.2680	0.3428	1.0140	0.052*	
C17	0.38181 (13)	0.38061 (12)	0.9246 (2)	0.0390 (4)	
H17	0.3685	0.4465	0.9160	0.047*	
N1	0.16655 (10)	0.42040 (10)	0.27386 (18)	0.0337 (3)	
N2	0.56126 (9)	0.20732 (9)	0.84612 (17)	0.0305 (3)	
H2	0.5741 (15)	0.1450 (8)	0.866 (3)	0.047 (6)*	
O1	0.22455 (12)	0.47889 (11)	0.24810 (18)	0.0545 (4)	
O2	0.13344 (13)	0.35960 (13)	0.1701 (2)	0.0684 (5)	
O3	0.33312 (7)	0.53639 (8)	0.57868 (15)	0.0318 (2)	
O4	0.22067 (8)	0.64521 (8)	0.49029 (15)	0.0326 (2)	
O5	0.09894 (10)	0.57856 (11)	0.93152 (18)	0.0465 (3)	
H5A	0.1152 (18)	0.6216 (15)	1.002 (3)	0.070*	
O6	0.23875 (9)	0.60562 (10)	0.86357 (17)	0.0435 (3)	
O7	0.43930 (9)	0.08565 (8)	0.94455 (17)	0.0378 (3)	
H7	0.3990 (13)	0.0519 (15)	0.981 (3)	0.057*	
O8	0.13275 (9)	0.78915 (10)	0.66412 (16)	0.0392 (3)	
H8A	0.1644 (15)	0.8061 (18)	0.7569 (18)	0.059*	
H8B	0.1616 (16)	0.7423 (13)	0.635 (3)	0.059*	

### Atomic displacement parameters (Å^2^)

**Table:**

	*U*^11^	*U*^22^	*U*^33^	*U*^12^	*U*^13^	*U*^23^
C1	0.0214 (5)	0.0215 (5)	0.0289 (6)	0.0023 (4)	0.0070 (5)	0.0021 (5)
C2	0.0244 (6)	0.0266 (6)	0.0286 (7)	0.0001 (5)	0.0074 (5)	0.0023 (5)
C3	0.0308 (7)	0.0365 (8)	0.0344 (8)	−0.0042 (6)	0.0123 (6)	0.0047 (6)
C4	0.0300 (7)	0.0364 (8)	0.0457 (9)	−0.0096 (6)	0.0101 (7)	0.0028 (7)
C5	0.0298 (7)	0.0321 (7)	0.0395 (8)	−0.0067 (6)	0.0064 (6)	−0.0041 (6)
C6	0.0244 (6)	0.0262 (6)	0.0304 (7)	0.0012 (5)	0.0076 (5)	−0.0007 (5)
C7	0.0269 (6)	0.0238 (6)	0.0262 (6)	−0.0013 (5)	0.0109 (5)	−0.0016 (5)
C8	0.0323 (7)	0.0301 (7)	0.0269 (7)	0.0003 (5)	0.0084 (5)	0.0024 (5)
C9	0.0337 (7)	0.0212 (6)	0.0294 (7)	0.0003 (5)	0.0014 (5)	0.0015 (5)
C10	0.0465 (9)	0.0239 (7)	0.0395 (8)	−0.0065 (6)	0.0006 (7)	0.0066 (6)
C11	0.0365 (8)	0.0441 (9)	0.0449 (10)	−0.0124 (7)	0.0041 (7)	0.0152 (8)
C12	0.0267 (7)	0.0467 (9)	0.0441 (9)	−0.0008 (6)	0.0088 (6)	0.0120 (7)
C13	0.0249 (6)	0.0221 (6)	0.0264 (6)	−0.0004 (4)	0.0034 (5)	0.0023 (5)
C14	0.0289 (7)	0.0256 (6)	0.0303 (7)	−0.0042 (5)	0.0052 (5)	0.0017 (5)
C15	0.0316 (7)	0.0420 (9)	0.0371 (8)	−0.0043 (6)	0.0103 (6)	0.0004 (7)
C16	0.0377 (8)	0.0510 (10)	0.0427 (9)	0.0119 (7)	0.0126 (7)	−0.0041 (8)
C17	0.0446 (9)	0.0305 (7)	0.0415 (9)	0.0111 (7)	0.0072 (7)	−0.0023 (7)
N1	0.0339 (7)	0.0353 (7)	0.0334 (7)	−0.0031 (5)	0.0103 (5)	−0.0067 (5)
N2	0.0281 (6)	0.0267 (6)	0.0368 (7)	0.0031 (4)	0.0069 (5)	0.0071 (5)
O1	0.0734 (10)	0.0545 (8)	0.0431 (7)	−0.0271 (7)	0.0301 (7)	−0.0107 (6)
O2	0.0782 (11)	0.0794 (12)	0.0555 (9)	−0.0402 (9)	0.0328 (8)	−0.0384 (8)
O3	0.0249 (5)	0.0287 (5)	0.0439 (6)	0.0004 (4)	0.0122 (4)	−0.0032 (4)
O4	0.0375 (6)	0.0241 (5)	0.0365 (6)	0.0007 (4)	0.0084 (5)	0.0031 (4)
O5	0.0434 (7)	0.0575 (8)	0.0442 (7)	−0.0089 (6)	0.0222 (6)	−0.0169 (6)
O6	0.0428 (7)	0.0496 (7)	0.0410 (7)	−0.0147 (6)	0.0153 (5)	−0.0122 (6)
O7	0.0393 (6)	0.0232 (5)	0.0537 (7)	−0.0050 (4)	0.0161 (5)	0.0044 (5)
O8	0.0429 (7)	0.0397 (7)	0.0361 (6)	0.0043 (5)	0.0104 (5)	0.0014 (5)

### Geometric parameters (Å, º)

**Table:**

C1—C6	1.392 (2)	C11—C12	1.383 (3)
C1—C2	1.4030 (19)	C11—H11	0.9300
C1—C7	1.5222 (18)	C12—N2	1.318 (2)
C2—C3	1.394 (2)	C12—H12	0.9300
C2—C8	1.489 (2)	C13—N2	1.3655 (18)
C3—C4	1.378 (2)	C13—C14	1.4097 (19)
C3—H3	0.9300	C14—O7	1.3437 (18)
C4—C5	1.377 (2)	C14—C15	1.362 (2)
C4—H4	0.9300	C15—C16	1.400 (3)
C5—C6	1.384 (2)	C15—H15	0.9300
C5—H5	0.9300	C16—C17	1.362 (3)
C6—N1	1.4654 (19)	C16—H16	0.9300
C7—O4	1.2404 (17)	C17—H17	0.9300
C7—O3	1.2577 (17)	N1—O1	1.2106 (18)
C8—O6	1.2028 (19)	N1—O2	1.2137 (19)
C8—O5	1.3142 (19)	N2—H2	0.889 (9)
C9—C10	1.403 (2)	O5—H5A	0.823 (10)
C9—C13	1.4085 (19)	O7—H7	0.841 (9)
C9—C17	1.410 (2)	O8—H8A	0.828 (10)
C10—C11	1.363 (3)	O8—H8B	0.829 (10)
C10—H10	0.9300		
			
C6—C1—C2	116.16 (12)	C10—C11—C12	119.32 (15)
C6—C1—C7	123.58 (12)	C10—C11—H11	120.3
C2—C1—C7	120.21 (12)	C12—C11—H11	120.3
C3—C2—C1	120.61 (14)	N2—C12—C11	120.37 (16)
C3—C2—C8	118.90 (13)	N2—C12—H12	119.8
C1—C2—C8	120.46 (12)	C11—C12—H12	119.8
C4—C3—C2	121.06 (14)	N2—C13—C9	119.16 (13)
C4—C3—H3	119.5	N2—C13—C14	119.89 (13)
C2—C3—H3	119.5	C9—C13—C14	120.94 (13)
C5—C4—C3	119.59 (14)	O7—C14—C15	126.44 (14)
C5—C4—H4	120.2	O7—C14—C13	115.32 (13)
C3—C4—H4	120.2	C15—C14—C13	118.23 (14)
C4—C5—C6	118.98 (15)	C14—C15—C16	121.17 (15)
C4—C5—H5	120.5	C14—C15—H15	119.4
C6—C5—H5	120.5	C16—C15—H15	119.4
C5—C6—C1	123.52 (14)	C17—C16—C15	121.51 (16)
C5—C6—N1	116.10 (13)	C17—C16—H16	119.2
C1—C6—N1	120.38 (12)	C15—C16—H16	119.2
O4—C7—O3	125.72 (13)	C16—C17—C9	119.08 (15)
O4—C7—C1	116.86 (12)	C16—C17—H17	120.5
O3—C7—C1	117.37 (12)	C9—C17—H17	120.5
O6—C8—O5	124.10 (15)	O1—N1—O2	122.38 (15)
O6—C8—C2	124.14 (14)	O1—N1—C6	119.10 (13)
O5—C8—C2	111.75 (13)	O2—N1—C6	118.52 (14)
C10—C9—C13	117.26 (14)	C12—N2—C13	122.68 (14)
C10—C9—C17	123.74 (14)	C12—N2—H2	119.6 (14)
C13—C9—C17	119.00 (14)	C13—N2—H2	117.5 (14)
C11—C10—C9	121.17 (15)	C8—O5—H5A	110.8 (19)
C11—C10—H10	119.4	C14—O7—H7	110.7 (17)
C9—C10—H10	119.4	H8A—O8—H8B	105 (2)
			
C6—C1—C2—C3	2.4 (2)	C9—C10—C11—C12	−1.1 (3)
C7—C1—C2—C3	−179.76 (13)	C10—C11—C12—N2	−0.1 (3)
C6—C1—C2—C8	−175.78 (13)	C10—C9—C13—N2	0.3 (2)
C7—C1—C2—C8	2.0 (2)	C17—C9—C13—N2	−179.84 (14)
C1—C2—C3—C4	0.0 (2)	C10—C9—C13—C14	−179.04 (14)
C8—C2—C3—C4	178.20 (15)	C17—C9—C13—C14	0.8 (2)
C2—C3—C4—C5	−2.1 (3)	N2—C13—C14—O7	−2.0 (2)
C3—C4—C5—C6	1.7 (3)	C9—C13—C14—O7	177.38 (14)
C4—C5—C6—C1	0.8 (2)	N2—C13—C14—C15	177.96 (14)
C4—C5—C6—N1	−178.24 (15)	C9—C13—C14—C15	−2.7 (2)
C2—C1—C6—C5	−2.9 (2)	O7—C14—C15—C16	−177.32 (17)
C7—C1—C6—C5	179.40 (14)	C13—C14—C15—C16	2.8 (2)
C2—C1—C6—N1	176.16 (13)	C14—C15—C16—C17	−1.0 (3)
C7—C1—C6—N1	−1.6 (2)	C15—C16—C17—C9	−1.0 (3)
C6—C1—C7—O4	96.24 (16)	C10—C9—C17—C16	−179.12 (17)
C2—C1—C7—O4	−81.38 (17)	C13—C9—C17—C16	1.0 (2)
C6—C1—C7—O3	−85.96 (18)	C5—C6—N1—O1	178.42 (16)
C2—C1—C7—O3	96.42 (16)	C1—C6—N1—O1	−0.7 (2)
C3—C2—C8—O6	157.79 (16)	C5—C6—N1—O2	−2.0 (2)
C1—C2—C8—O6	−24.0 (2)	C1—C6—N1—O2	178.93 (17)
C3—C2—C8—O5	−23.5 (2)	C11—C12—N2—C13	1.5 (3)
C1—C2—C8—O5	154.77 (14)	C9—C13—N2—C12	−1.6 (2)
C13—C9—C10—C11	1.0 (2)	C14—C13—N2—C12	177.75 (15)
C17—C9—C10—C11	−178.83 (17)		

### Hydrogen-bond geometry (Å, º)

**Table:**

*D*—H···*A*	*D*—H	H···*A*	*D*···*A*	*D*—H···*A*
N2—H2···O3^i^	0.89 (1)	2.00 (1)	2.8112 (16)	151 (2)
O5—H5*A*···O8^ii^	0.82 (1)	1.78 (1)	2.5928 (18)	171 (3)
O7—H7···O3^iii^	0.84 (1)	1.82 (1)	2.6482 (15)	168 (2)
O8—H8*B*···O4	0.83 (1)	2.07 (1)	2.8683 (17)	163 (2)
O8—H8*A*···O4^ii^	0.83 (1)	2.01 (1)	2.8288 (18)	170 (2)
C11—H11···O1^iv^	0.93	2.42	3.295 (2)	156
C12—H12···O6^i^	0.93	2.48	3.343 (2)	155
C16—H16···O2^v^	0.93	2.52	3.413 (2)	160

Symmetry codes: (i) −*x*+1, *y*−1/2, −*z*+3/2; (ii) *x*, −*y*+3/2, *z*+1/2; (iii) *x*, −*y*+1/2, *z*+1/2; (iv) −*x*+1, −*y*+1, −*z*+1; (v) *x*, *y*, *z*+1.
