# Supplementary figures and images for: Crystal structure of 8-hy­droxy­quinolin­ium 2-carboxy-6-nitro­benzoate mono­hydrate
Source: Acta Crystallogr E Crystallogr Commun. 2015 Mar 25;71(Pt 4):o261–2. doi: 10.1107/S205698901500571X (PMC4438817; doi:10.1107/S205698901500571X)

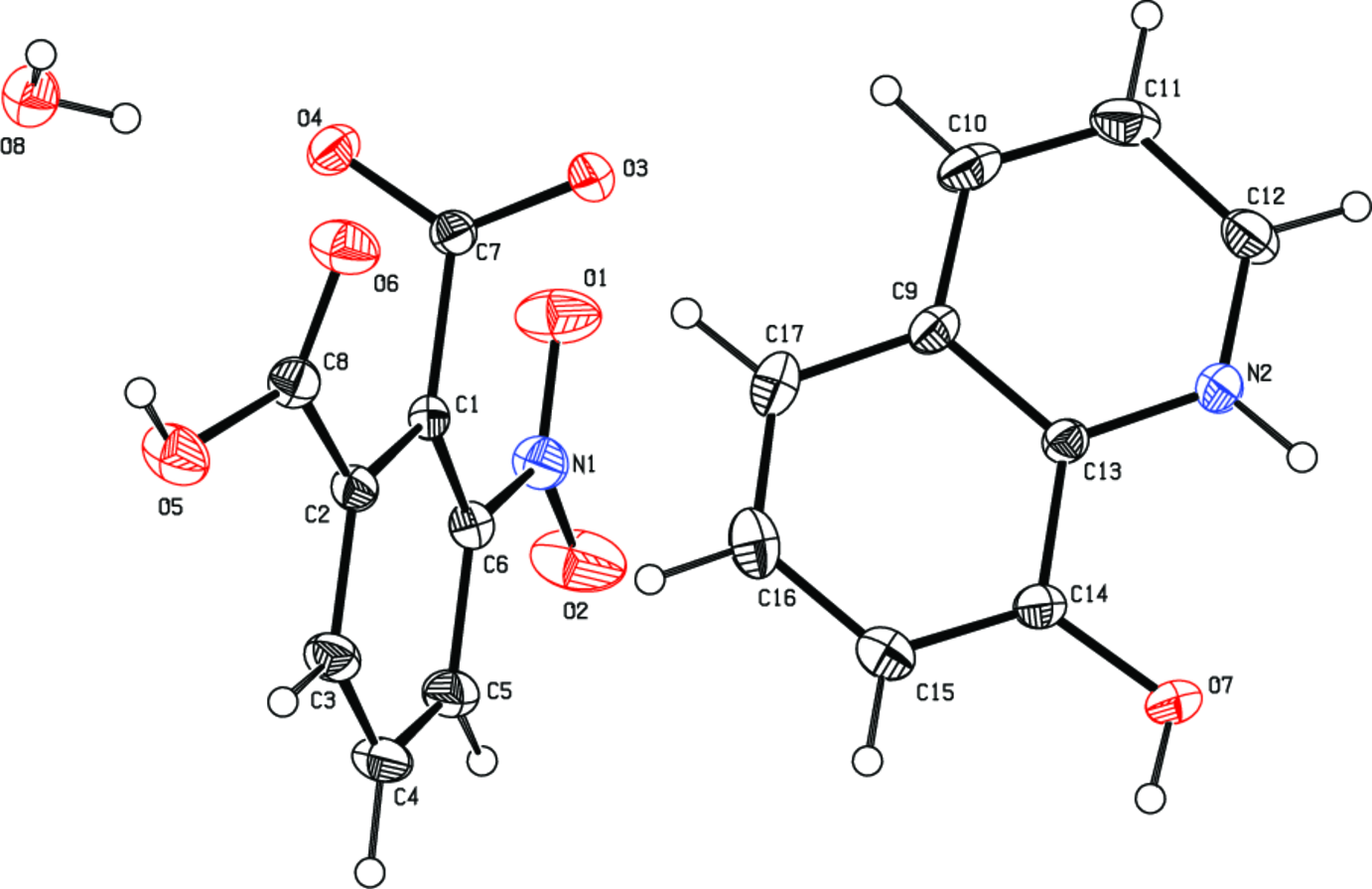

Supplement: Supplementary file 4 [file e-71-0o261-fig1.tif]

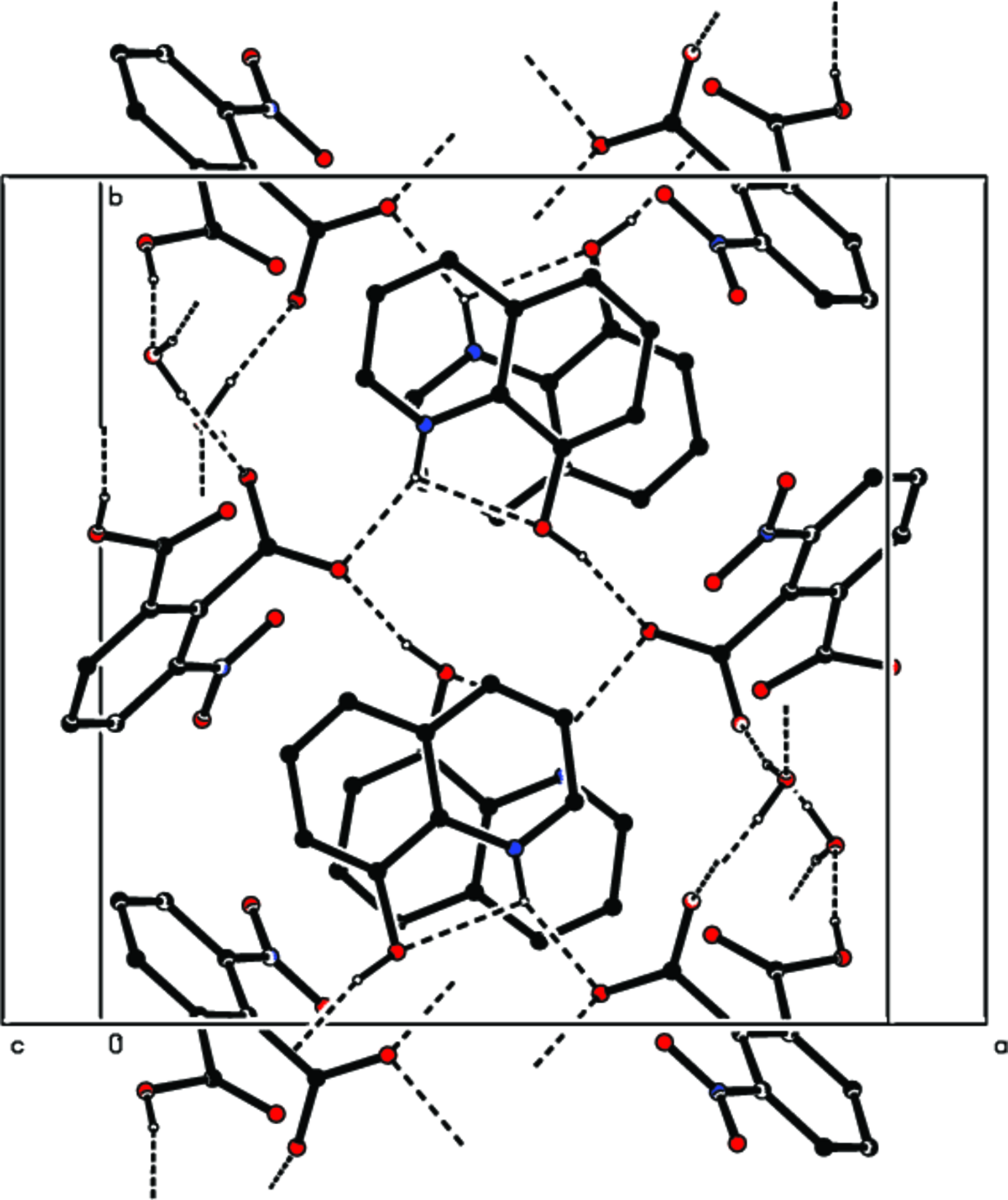

Supplement: Supplementary file 5 [file e-71-0o261-fig2.tif]
